# High efficacy of artemether–lumefantrine and dihydroartemisinin–piperaquine for the treatment of uncomplicated falciparum malaria in Muheza and Kigoma Districts, Tanzania

**DOI:** 10.1186/s12936-018-2409-z

**Published:** 2018-07-11

**Authors:** Celine I. Mandara, Reginald A. Kavishe, Samuel Gesase, Janneth Mghamba, Esther Ngadaya, Peter Mmbuji, Sigsbert Mkude, Renata Mandike, Ritha Njau, Ally Mohamed, Martha M. Lemnge, Marian Warsame, Deus S. Ishengoma

**Affiliations:** 1 National Institute for Medical Research, Tanga Centre, Tanga, Tanzania; 2 Kilimanjaro Christian Medical University College, Moshi, Tanzania; 3 Epidemiology and Disease Control Section, Ministry of Health, Community Development, Gender, Elderly and Children, Dar es Salaam, Tanzania; 4 National Institute for Medical Research, Muhimbili Centre, Dar es Salaam, Tanzania; 5 National Malaria Control Program, Dar es Salaam, Tanzania; 6 World Health Organization Country Office, Dar es Salaam, Tanzania; 7 World Health Organization, Geneva, Switzerland

**Keywords:** Efficacy, Parasite clearance, Artemether–lumefantrine, Dihydroartemisinin–piperaquine, *Plasmodium falciparum*

## Abstract

**Background:**

Artemether–lumefantrine (AL) is the recommended first-line artemisinin-based combination therapy (ACT) for the treatment of uncomplicated falciparum malaria in most of the malaria-endemic countries, including Tanzania. Recently, dihydroartemisinin–piperaquine (DP) has been recommended as the alternative anti-malarial to ensure effective case management in Tanzania. This study assessed the parasite clearance rate and efficacy of AL and DP among patients aged 6 months to 10 years with uncomplicated falciparum malaria in two sites with different malaria transmission intensity.

**Methods:**

This was an open-label, randomized trial that was conducted at two sites of Muheza Designated District Hospital and Ujiji Health Centre in Tanga and Kigoma regions, respectively. Patients meeting inclusion criteria were enrolled, treated with either AL or DP and followed up for 28 (extended to 42) and 42 (63) days for AL and DP, respectively. Parasite clearance time was monitored in the first 72 h post treatment and the clearance rate constant and half-life were calculated using an established parasite clearance estimator. The primary outcome was parasitological cure on days 28 and 42 for AL and DP, respectively, while secondary outcome was extended parasitological cure on days 42 and 63 for AL and DP, respectively.

**Results:**

Of the 509 children enrolled (192 at Muheza and 317 at Ujiji), there was no early treatment failure and PCR uncorrected cure rates on day 28 in the AL group were 77.2 and 71.2% at Muheza and Ujiji, respectively. In the DP arm, the PCR uncorrected cure rate on day 42 was 73.6% at Muheza and 72.5% at Ujiji. With extended follow-up (to day 42 for AL and 63 for DP) cure rates were lower at Ujiji compared to Muheza (AL: 60.2 and 46.1%, p = 0.063; DP: 57.6 and 40.3% in Muheza and Ujiji, respectively, p = 0.021). The PCR corrected cure rate ranged from 94.6 to 100% for all the treatment groups at both sites. Parasite clearance rate constant was similar in the two groups and at both sites (< 0.28/h); the slope half-life was < 3.0 h and all but only one patient cleared parasites by 72 h.

**Conclusion:**

These findings confirm high efficacy of the first- and the newly recommended alternative ACT for treatments for uncomplicated falciparum malaria in Tanzania. The high parasite clearance rate suggests absence of suspected artemisinin resistance, defined as delayed parasite clearance.

*Trial registration* This trial is registered at ClinicalTrials.gov under registration number NCT02590627

## Background

Effective case management based on early diagnosis and prompt treatment with effective drugs has been one of the core malaria interventions [1]. Despite reports of declining burden of malaria attributed to intensified scale-up of interventions, the disease is still a leading cause of morbidity and mortality with estimated 216 million cases and 446,000 deaths in 2016, mostly among under-fives and pregnant women from sub-Saharan Africa [1]. Recent studies have shown that artemisinin-based combination therapy (ACT) is still efficacious and safe for the treatment of uncomplicated falciparum malaria especially in Africa [1, 2], but emergence of resistant *Plasmodium falciparum* has been reported in Southeast Asian (SEA) countries [3–5] and might reverse the recent gains in malaria control, and jeopardize malaria elimination efforts. The World Health Organization (WHO) recommends that malaria-endemic countries should monitor the efficacy of nationally recommended ACT in order to guide national treatment guidelines [2].

Artemisinin-based combinations recommended by WHO includes artemether–lumefantrine (AL), artesunate–amodiaquine (AS + AQ), artesunate–mefloquine (AS + MQ), artesunate–sulphadoxine/pyrimethamine (AS + SP), and dihydroartemisinin–piperaquine (DP) [6]. Of these, AL is the most widely used combination and it is currently the first-line anti-malarial drug in most malaria-endemic countries in the WHO African region [7]. Due to high level of resistance to SP, Tanzania adopted AL in 2006 and the implementation of the new policy started in January 2007 [8]. DP was recommended in 2014 as an alternative drug for the treatment of uncomplicated falciparum malaria in Tanzania in order to improve case management [9]. Baseline data on the efficacy of DP is urgently needed to support future surveillance of its performance once it has been widely used in the country.

Following the WHO recommendation to monitor the efficacy of ACT every 2 years, Tanzania has been conducting series of studies under the national malaria control programme (NMCP) framework to test for efficacy and safety of ACT, particularly after policy changes in 2006 [10]. Studies conducted in Tanzania [10, 11] and elsewhere have showed that AL is still efficacious (91–100%) and highly tolerated when used for the treatment of uncomplicated falciparum malaria [12–18]. For DP, studies conducted in Uganda [19], Kenya [16] and Mozambique [12] have reported high cure rates and safety profile of the drug with longer post-treatment prophylactic effect due to longer half-life of piperaquine compared to lumefantrine. However, DP was recently introduced in Tanzania as an alternative anti-malarial for treatment of uncomplicated falciparum malaria when indicated and thus, its efficacy and safety need to also be assessed regularly as recommended by WHO. Intensified surveillance of anti-malarial efficacy and safety of AL and DP is urgently needed to monitor their performance.

In addition to proportion of treatment failure, therapeutic efficacy studies also generate information on parasite clearance in terms of proportion of day 3 parasite positivity or [20] half-life of the parasite clearance slope, estimated through multiple daily parasitaemia in the first 72 h post-treatment (e.g., at 6, 8 or 12 hourly interval) [21, 22]. Most of the studies conducted in Tanzania [10] and elsewhere in Africa [13, 23] have showed that day 3 positivity rate is very low (< 2.0%) which is below the WHO cut-off point of 10%, above which artemisinin resistance is suspected [20]. The few studies incorporating regular parasite sampling conducted in Mali [24], Kenya [16] and Uganda [23] have not reported any signs of delayed parasite clearance, but further surveillance is required to longitudinally monitor and report any occurrence of suspected tolerance/resistance to artemisinins, particularly with continued use of these drugs in Africa. The present study was conducted at two NMCP sentinel sites of Muheza and Kigoma districts in Tanga and Kigoma regions, respectively, and incorporated frequent parasite sampling to assess parasite clearance time and in vivo efficacy of AL and DP for the treatment of uncomplicated falciparum malaria. The data generated provide critical information on efficacy, including parasite clearance time of both DP and AL in Tanzania and will inform the national treatment policy.

## Methods

### Study area

The study was conducted between May 2014 and January 2015, at Muheza Designated District Hospital in Muheza, Tanga region, and Ujiji Health Centre (Kigoma region) in Northeastern and Western Tanzania, respectively. These sites are among the eight NMCP sentinel sites for monitoring the efficacy of anti-malarial drugs in Tanzania, and were classified as holoendemic (Muheza) and mesoendemic (Ujiji) for malaria in 1990s [25, 26]. However, malaria transmission has changed in recent years with Muheza becoming a low transmission while Ujiji has persistently remained a moderate transmission area [27–29]. A detailed description of the study sites has been provided elsewhere [30].

### Study design and target population

This was an open-label, randomized trial that assessed the efficacy of AL and DP for the treatment of uncomplicated falciparum malaria. Patients aged 6 months to 10 years were recruited at the outpatient departments (OPDs) and assessed for inclusion in the study based on the WHO protocol of 2009 [31].

### Sample size estimation

The sample size was calculated to test the hypothesis that the risk of treatment failure (adjusted by PCR genotyping to distinguish recrudescent from new infections) after day 28, 42 or 63 would not differ between the AL and DP treated children. Based on previous data from areas of high malaria transmission intensity which showed that the efficacy (adjusted by PCR genotyping) after 42 days was estimated to be 98% after treatment with DP and 92% for patients treated with AL [13], 125 patients were targeted in each treatment arm at each site. The estimated sample size was adjusted to allow for 20% loss to follow-up and withdrawals giving a total sample size of 150 patients per treatment arm per site.

### Screening and randomization of study participants

Children aged 6 months to 10 years from OPDs of Muheza hospital and Ujiji Health Centre were screened initially with rapid diagnostic tests for malaria (RDTs) and confirmed by microscopy as previously described [30]. Patients meeting inclusion criteria as per WHO protocol of 2009 [31] with minor modifications (to assess cardiotoxicity effects associated with DP [32]) were recruited and randomized to receive therapeutic doses of either AL or DP. The inclusion criteria among others were a microscopically confirmed mono-infection of *P. falciparum*, and parasitaemia between 250 and 200,000 asexual parasites/µl of blood, axillary temperature ≥ 37.5 °C or a history of fever within the past 24 h and ability to swallow oral medications. Others included the ability and willingness to attend scheduled follow-up visits, providing an informed consent by parent or guardian and stable residence within the catchment areas of the study health facilities throughout the study period.

Exclusion criteria were severe malnutrition, febrile conditions due to diseases other than malaria, presence of danger signs due to severe falciparum malaria, severe anaemia (Hb < 5 g/dl), mixed or mono-infections with other *Plasmodium* species, severe diarrhoea with dehydration and use of regular medications which could interfere with anti-malarial pharmacokinetics. Patients with a history of hypersensitivity reactions or contra-indications to ACT were also excluded. Assessment was also done to exclude patients taking medicinal products known to prolong QTc interval and treatment with DP in the previous 4 weeks, but not with AL. Patients meeting any of the exclusion criteria were excluded from the study but were treated according to the national guidelines [8].

A randomization list was computer generated for different age-strata (< 2 years; 2 to < 5 years and ≥ 5 to 10 years) using Microsoft Excel. Sequentially numbered, sealed envelopes containing the treatment group assignments were prepared from the randomization list for each age category. These numbers were used to assign patients to the treatment arms based on their age groups, with a target of getting equal numbers of children aged < 2 years, 2 to < 5 years and ≥ 5 to 10 years.

### Treatment and follow-up

All eligible patients were randomized to receive either AL, a fixed combination of 20 mg artemether and 120 mg lumefantrine in a tablet (Coartem^®^, IPCa Laboratories Ltd, Kandivil, Mumbai, India) or DP, a fixed combination of 20 mg dihydroartemisinin and 160 mg piperaquine (Duo-cotexin^®^, Holley Pharm, PR China). The drugs were administered orally under supervision of the study nurse based on patients’ weight. For AL: 1 tablet was given to children weighing 5–14 kg, 2 tablets to those weighing 15–24 kg and 3 tablets to children weighing 25–34 kg. A full course of AL consisted of 6 doses given twice daily (8 hourly apart on day 0 and 12 hourly apart on days 1 and 2). DP was given according to body weight; half a tablet was administered to children weighing 5 to < 7 kg, 1 tablet to those with 7 to < 13 kg, 2 tablets to those weighing 13 to < 24 kg, and 3 tablets for those with 24 to < 36 kg. A full course of DP consisted of three equally divided doses given once daily at an interval of 24 h apart. Patients were observed for 30 min post-treatment; if vomiting occurred within 30 min, a second full dose was repeated. Persistent vomiting of the second dose led to withdrawal from the study and, administration of rescue medicine, with parenteral quinine or injectable artesunate according to the national guidelines for management of complicated and severe malaria [8]. Paracetamol was also given to all patients with body temperature ≥37.5 °C.

Patients were admitted at the health facility for the first 3 days to ensure strict follow-up and adherence to dosing intervals. It also allowed 8 hourly blood slide collection for assessment of parasite clearance until two successive blood smears turned out to be negative. Even after complete clearance of the parasites, patients were retained at the clinic to complete the treatment and collection of the day 3 blood smears before they could be discharged. Patients were seen by study clinicians on days 0, 1, 2, and 3 and then discharged home to attend the weekly follow-up visits from day 7 to day 42 for AL, and day 63 for DP. Parents/care-takers were advised and encouraged to bring back their children to the study clinic at any other time (unscheduled visits) if they felt unwell. During these visits, study clinicians collected medical history, vital signs, malaria blood smears, and adverse events using standard questionnaire guides. Patients who could not attend the scheduled visits by mid-day were traced at their home by a member of the study team and brought to the facility. Those who travelled to other areas and could not be traced for their scheduled follow-up were classified as loss to follow-up and withdrawn from the study.

### Sample collection and processing

On the enrolment day, finger-prick blood was initially collected after parents’/guardians’ consent to confirm parasitaemia by RDT and microscopy as earlier described [30]. For patients who were enrolled in the study: venous blood samples (5–7 ml) were collected on the day of enrolment for malaria parasite identification, genomic studies of parasites and human, measurement of haemoglobin levels and preparation of blood spots on filter papers (Whatman No. 3, GE Healthcare Bio-Sciences, PA, USA). During follow-up, blood smears and filter-paper blood spots were prepared from a finger prick. All filter-paper blood samples were air-dried and stored in zip lock envelopes with desiccators for further analysis. Further processing of parasites and genomic analysis targeting parasites and human genes are underway and the findings will be reported elsewhere.

### Microscopic diagnosis of malaria parasites

For patients with positive RDT results, two blood smears (thick and thin) were prepared through finger prick at screening and 1 slide was stained with 10% Giemsa for 10–15 min for initial assessment of eligibility to participate in the study, while the second smear was retained. For enrolled patients, the second slide was stained carefully (with 2.5–3% Giemsa stain for 45–60 min) for accurate counting of malaria parasites and detection of parasite species and gametocytes. Similarly, slower staining was used for all slides that were collected at 8-h intervals for assessment of parasite clearance in the first 72 h and during weekly follow-up visits. The thick blood smear for initial screening was used to count the numbers of asexual parasites and white blood cells in a limited number of microscopic fields to determine if the patient was eligible for enrolment. Each of the second blood smear and smears collected during follow-up were examined by two independent microscopists and parasites were counted as asexual parasites per 200 white blood cells (WBCs) while sexual parasites were accounted per 500 WBCs. Parasite density was calculated by multiplying the number of asexual parasites by 40 and 16 for asexual and sexual parasites, respectively; assuming that 1 µl of blood contained 8000 WBCs [33]. A blood slide was declared negative when examination of 100 high power fields did not reveal the presence of any malaria parasite. Blood smears with discordant results (differences between the two microscopists in species diagnosis, presence of parasites or parasite density of > 50%) were re-examined by a third, independent microscopist, and parasite density was calculated by averaging the two closest counts.

### Genotyping of malaria parasites

In order to distinguish recrudescence from newly acquired infections, venous blood (3–7 ml) was collected from all study patients on day 0 (before administration of study drugs) and filter paper (Whatman No. 3) blood samples were collected on day 7 and onwards. Parasite DNA was extracted from venous blood or dried blood spots (DBS) using QIAamp DNA blood midi kits (QiAgen GmbH, Hilden, Germany) according to the manufacturer’s instructions. Paired DNA samples (day 0 and day of parasites recurrence) were genotyped by analysing the polymorphic loci of merozoite surface proteins 1 and 2 (*msp1* and *msp2*), and glutamate rich protein (*glurp*) genes according to the standard protocol [31, 34].

### Haematological assessment

A portion of venous blood samples collected on day 0 was used to measure haemoglobin (Hb) levels (g/dl) using a Haemocue^®^ machine (HemoCue, Ångelholm, Sweden). The Hb was also measured using finger prick blood during weekly follow-up visits from day 14 to 42 for AL or day 14 to 63 for DP.

### Outcome classification

The primary endpoint was adequate clinical and parasitological response (ACPR) on day 28 for AL, and day 42 for DP as per WHO protocol [31]. Secondary endpoints included extended parasitological cure on day 42 and day 63 for AL and DP, respectively, parasite clearance by 72 h, improvement in haemoglobin levels comparing baseline and follow-up visits and reduction in gametocyte carriage during follow-up compared to day 0 baseline.

Treatment outcomes were classified as per WHO protocol of 2009 either as early treatment failure (ETF), late clinical failure (LCF), late parasitological failure (LPF), or adequate clinical and parasitological response (ACPR) [31].

### Ethical considerations

The study protocol was reviewed and approved by the Tanzanian Medical Research coordinating Committee (MRCC) of the National Institute for Medical Research (NIMR). Permission to conduct the study in Muheza–Tanga and Ujiji–Kigoma was sought in writing and obtained from the district and regional medical officers. Written informed consent was obtained from children’s parents or guardians before screening. Appropriate information (about the study and the protocol/methods) in a language that was understood by the parents/guardians of the study patients was compiled and provided before consent was obtained.

### Data management and analysis

The data was double-entered into a Microsoft Access database followed by validation, cleaning and analysis using STATA version 11 (STATA Corporation, TX-USA). Descriptive statistics (mean, standard deviation and proportions) was used to describe the study population and present treatment outcomes. The data were also transferred to the WHO Excel software programme [35], for automatic analysis of treatment outcomes. Differences in proportions of treatment outcomes within and between the two study sites were compared using Chi squared test. Student’s t test (for continuous variables) or Mann–Whitney *U* test (a non-parametric test for non-normally distributed data) were applied for analysis of continuous variables such as parasite density, age and Hb levels. Analysis was performed based on per protocol method and Kaplan–Meier survival analysis. Analysis of parasite clearance was performed using the parasite clearance estimator as previously described [21, 22]. The minimum detectable parasitaemia was set at 16 asexual parasite/µl of blood. During the analysis of parasite clearance (0–72 h), samples with too few data points and/or too low parasitaemia were excluded. Different estimates were generated including clearance rate constant (estimated clearance rate constant/h), slope half-life (estimated time in hours for parasitaemia to decline by half), and P50 and P95 (estimated time in hours for the parasitaemia to decline by 50 and 95% of the initial values, respectively). p values < 0.05 were considered statistically significant.

## Results

### Trial profile and baseline characteristics

**Fig. 1:**
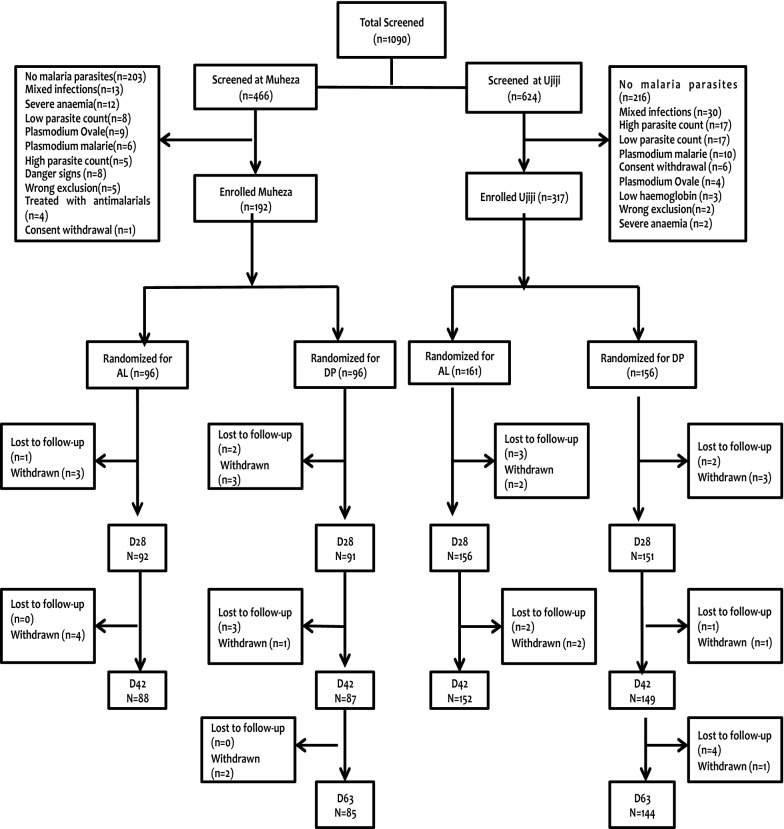
Trial profile showing screening, randomization and follow-up of children treated with either AL or DP at Muheza and Ujiji sites

Overall, 1090 patients were screened, 671 patients (61.5%) were positive for *P. falciparum* and 509 were recruited at both sites of Muheza (n = 192) and Ujiji (n = 317). Of these, 257 (50.5%) were randomized to receive AL and 252 (49.5%) received DP. Twenty-two patients (4.3%) were excluded from the study due to various reasons while 18 (3.5%) were lost to follow-up (Fig. 1). Within each site, most of the baseline characteristics were similar in the 2 groups (Table 1). However, children recruited at Ujiji had significantly higher mean age (p < 0.001), haemoglobin concentration (p < 0.001) and parasitaemia (p < 0.001). The proportion of male patients recruited at Muheza was higher compared to Ujiji (p = 0.029) (Table 1).

**Table 1:** Baseline characteristics of children enrolled at Muheza and Ujiji

Variable	Muheza		Ujiji	
	AL (n = 96)	DP (n = 96)	AL (n = 161)	DP (n = 156)
Age in years, mean (SD)	3.3 (2.2)	3.1 (2.0)	3.8 (2.4)	4 (2.6)
Weight (kg), mean (SD)	13.3 (4.4)	13.1 (4.4)	13.8 (4.3)	13.7 (4.2)
Gender (male), n (%)	59 (61.5)	56 (58.3)	83 (51.6)	77 (49.4)
Body temperature in °C, mean (SD)	38.6 (1.1)	38.6 (1.2)	38.1 (1.3)	38.1 (1.4)
Parasitaemia (µl) GMPD (counts/μl))	29,132	35,317	56,018	47,535
Hb in g/dl, mean (SD)	8.9 (1.7)	8.9 (1.6)	9.5 (1.7)	9.6 (1.6)

dl, decilitre; g, gram; GMPD,  geometric mean parasite density; Hb, haemoglobin; kg, kilogram; n, number of patients; SD, standard deviation; °C, degrees Celsius; %, percentage

### Treatment outcome

There was no ETF, however cases with recurrent infections increased at the two sites and within the treatment groups as the follow up was extended (Table 2). Among patients treated with AL, uncorrected ACPR on day 28 was slightly higher at Muheza compared to Ujiji (77.2 and 71.2%, respectively). However, when the follow up was extended to 42 days, uncorrected ACPR was lower at Ujiji compared to Muheza (46.1 and 60.2%, respectively), although the difference was not statistically significant (p = 0.063). Similarly, the uncorrected ACPR among patients treated with DP was not significantly different at the two sites (73.6% at Muheza and 72.5% at Ujiji) during the 42 days of follow-up, but it was significantly lower at Ujiji compared to Muheza when the follow-up was extended to 63 days (40.3 and 57.6%, respectively; p = 0.021) (Table 2). However, when the treatment outcomes were adjusted by PCR, the cure rate of the two drugs was similar, and ranged from 95.1 to 100% at different follow-up points in both sites (Table 2).

**Table 2:** Treatment outcome of cases treated with either artemether–lumefantrine or dihydroartemisinin at Muheza and Ujiji

Outcome	Muheza—AL (n = 96)				Ujiji—AL (N = 161)			
	Day 28 follow-up		Day 42 follow-up		Day 28 follow-up		Day 42 follow-up	
	PCR uncorrected	PCR corrected	PCR uncorrected	PCR corrected	PCR uncorrected	PCR corrected	PCR uncorrected	PCR corrected
ETF	0 (0.0)	0 (0.0)	0 (0.0)	0 (0.0)	0 (0.0)	0 (0.0)	0 (0.0)	0 (0.0)
LCF	11 (12.0)	0 (0.0)	19 (21.6)	0 (0.0)	15 (9.6)	3 (2.6)	23 (15.1)	3 (4.1)
LPF	10 (10.9)	0 (0.0)	16 (18.2)	0 (0.0)	30 (19.2)	0 (0.0)	59 (38.8)	1 (1.4)
ACPR	71 (77.2)	71 (100.0)	53 (60.2)	53 (100.0)	111 (71.2)	111 (97.4)	70 (46.1)	70 (94.6)
WTH/LFU	4 (4.2)	25 (26.0)	8 (8.3)	40 (43.0)	5 (3.1)	44 (27.8)	9 (5.6)	82 (52.6)
PCR ND	NA	0	NA	3 (3.1)	NA	3 (1.9)	NA	5 (3.1)

Outcome	MUHEZA—DP (96)				UJIJI—DP (N = 156)			
	Day 42 follow-up		Day 63 follow-up		Day 42 follow-up		Day 63 follow-up	
	PCR uncorrected	PCR corrected	PCR uncorrected	PCR corrected	PCR uncorrected	PCR corrected	PCR uncorrected	PCR corrected
ETF	0 (0.0)	0 (0.0)	0 (0.0)	0 (0.0)	0 (0.0)	0 (0.0)	0 (0.0)	0 (0.0)
LCF	4 (4.6)	0 (0.0)	17 (20.0)	0 (0.0)	11 (7.4)	1 (0.9)	31 (21.5))	1 (1.6)
LPF	19 (21.8)	2 (3.0)	19 (22.4)	3 (5.8)	30 (20.1)	0 (0.0)	55 (38.2)	2 (3.3)
ACPR	64 (73.6)	64 (97.0)	49 (57.6)	49 (94.2)	108 (72.5)	108 (99.1)	58 (40.3)	58 (95.1)
WTH/LFU	9 (9.4)	25 (27.5)	11 (11.5)	38 (42.2)	7 (4.5)	46 (29.7)	12 (7.7)	90 (59.6)
PCR ND	NA	5 (5.2)	NA	6 (6.3)	NA	1 (0.6)	NA	5 (3.2)

*ACPR* adequate clinical and parasitological response, *AL* artemether–lumefantrine, *DP* dihydroartemisinin–piperaquine, *ETF* early treatment failure, *LCF* late clinical failure, *LFU* lost to follow-up, *LPF* late parasitological failure, *NA* not applicable, *PCR* polymerase chain reaction, *PCR ND* PCR not done, *WTH* withdrawn

### Parasite clearance

A total of 35 individuals were excluded in the analysis of parasite clearance leaving 474 patients. The rates of parasite clearance based on different estimates were similar for both drugs and at both sites (Table 3). However, the slope half-life was slightly higher at Muheza compared to Ujiji in both treatment groups and all patients had cleared parasites by day 3 except one patient in the AL arm (1.1%) at Muheza (Table 4).

**Table 3:** Parasite clearance within 72 h based blood smears collected after every 8 h

Item	Muheza—AL	Ujiji—AL	Muheza—DP	Ujiji—DP
Clearance rate constant (IQR) (/h)	0.23	0.25	0.22	0.28
	(0.20, 0.28)	(0.21, 0.31)	(0.19, 0.27)	(0.23, 0.37)
Slope half-life (h)	2.96	2.75	3.09	2.51
	(2.52, 3.52)	(2.22, 3.27)	(2.58, 3.64)	(1.85, 3.05)
P50 (h)^a^	11.22	8.54	6.81	6.08
	(6.89, 14.00)	(5.37, 12.11)	(4.35, 10.60)	(3.77, 10.19)
P95 (h)^b^	20.34	17.49	17.4	14.76
	(17.08, 23.43)	(14.30, 21.06)	(13.47, 20.43)	(12.36, 17.75)

^a^Estimated time in hours for parasitaemia to decline by 50% of the initial values

^b^Estimated time in hours for parasitaemia to decline by 95% of the initial values

**Table 4:** Parasite clearance between day 0 and 3 based on 24 h sampling

Variable	AL	DP	Overall
Muheza			
With *P. falciparum*			
Day 1	86/95 (90.5%)	86/96 (89.6%)	172/191 (90.1%)
Day 2	21/94 (22.3%)	21/96 (21.9%)	42/190 (22.1%)
Day 3	1/94 (1.1%)	0 (0.0%)	1/189 (0.5%)
Ujiji			
With *P. falciparum*			
Day 1	137/161 (85.1%)	117/155 (75.5%)	254/316 (80.4%)
Day 2	35/159 (22.0%)	17/154 (11.0%)	52/113 (16.6%)
Day 3	0 (0.0%)	0 (0.0%)	0 (0.0%)

### Gametocyte clearance

Gametocyte carriage by microscopy was higher at Muheza where individuals with gametocytes increased between days 1 and 2 and then declined on day 3, with complete clearance by day 7. Low gametocyte carriage was observed at Ujiji where only one patient treated with AL had gametocytes on days 1 and 2 and total clearance was attained by day 3 (Table 4).

### Haemoglobin recovery

**Fig. 2:**
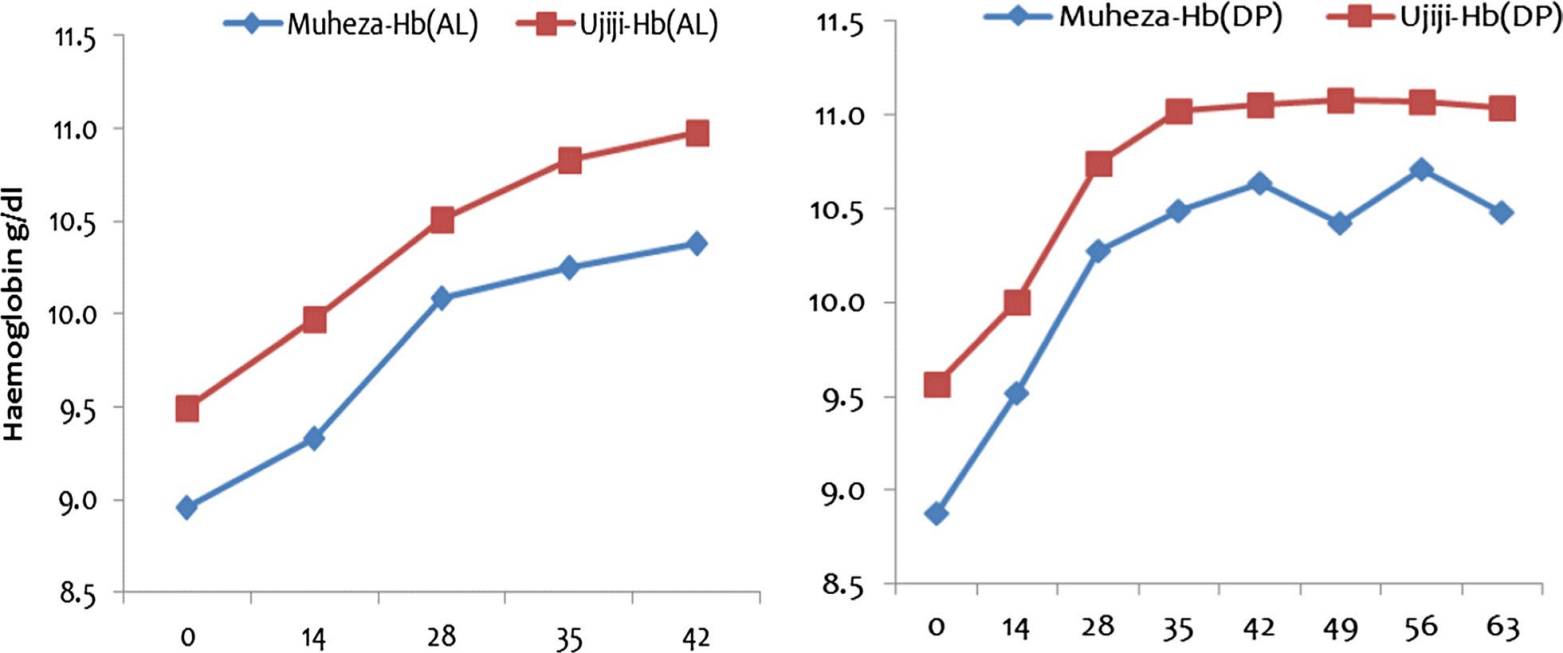
Haemoglobin levels of children treated with AL or DP measured at enrolment on day 0 to day 42 (AL) and day 0 to ay 63 (DP) at Muheza and Ujiji sites

At both sites, a highly significant increase in mean Hb (g/dl) was observed between day 0 and day 28 in the AL arm (p < 0.001) (Fig. 2). With extended follow-up, the mean Hb increased further at Muheza but significant changes were reported at Ujiji (p < 0.001). For the patients treated with DP (at both sites), the mean Hb increased between day 0 and day 28 while no significant changes were seen at Muheza and a slight decrease was reported at Ujiji during extended follow-up to day 63 (Fig. 2).

## Discussion

The findings of this study showed that both AL and DP are highly efficacious for the treatment of uncomplicated falciparum malaria in the study areas; with PCR corrected ACPR of 97.4–100% on day 28 for AL and 97.0–99.1% on day 42 for DP. The observed high efficacy of AL was similar to that of other studies from different parts of Tanzania [10, 11] and elsewhere in Africa [12–18], where similar PCR corrected cure rates were reported supporting the maintained high efficacy of AL despite its use in Africa for more than a decade. However, studies done in Angola in 2013 and 2015 reported low efficacy of AL (PCR corrected cure rate of 88%) at one of the sites [36, 37] and that is lower compared to cure rates reported in the majority of studies that were conducted in the sub-Saharan-African region [12–18]. However, patients enrolled in both Angolan studies took the evening doses of AL at home unsupervised, and this might explain the high treatment failure rate.

This study also showed high PCR corrected ACPR of DP, which is consistent with the findings from recent studies conducted in Tanzania (Kakolwa et al. manuscript in preparation) and other African countries [16, 18, 19, 36, 38, 39]. DP has been deployed as an alternative ACT for the treatment of uncomplicated falciparum malaria in mainland Tanzania since 2014. The finding of PCR corrected cure rate > 97% with DP treatment (during the 42 days of follow-up) supports the decision made by NMCP and is also in line with WHO recommendation that an ACT should have cure rate of > 95% before including it in the treatment policy [6]. The rapid development and spread of piperaquine resistance in SEA, leading to high treatment failure with DP [4, 40], underscores the need to closely monitor the efficacy of DP and molecular marker associated with piperaquine resistance.

There were high and similar parasite recurrences in both sites, which were due to re-infection as confirmed by PCR analysis. However, re-infection rate during extended follow-up was higher at Ujiji site for both drugs, possibly related to relatively higher malaria transmission in this site compared to Muheza [27–29]. From operational perspective, WHO recommends to consider parasite recurrence before 28 days of treatment with ACT as recrudescence (true treatment failure) and they should be treated with the second line anti-malarial; those which occur after 28 days as new infections and should be treated with the same drug [6]. However, efforts should be directed at intensifying malaria control so that the risk of malaria infection in the community can be further reduced or eliminated.

According to WHO [20], delayed parasite clearance (slope half-life > 5 h or day 3 positivity rate > 10%) indicate suspected artemisinin resistance. This study showed that both drugs had fast parasite clearance in terms of parasite clearance rate constant and slope half-life as well as day 3 positivity rates, indicating absence of suspected artemisinin resistance. Similar findings have also been reported from other African countries [16, 23, 24] suggesting that artemisinin resistance has not emerged in Africa. In addition, molecular studies in Africa have showed absence of the known mutations in the kelcher 13 (*k*-*13*) *gene* associated with artemisinin resistance in SEA [41–43], further suggesting that artemisinins are still effective and their parasite clearance capacity has not been altered. However, due to the spread of artemisinin resistance in SEA [3–5, 20], African countries have to be vigilant about its potential emergence through continuous monitoring of the efficacy and parasite clearance of ACT, and surveillance of polymorphism in the *k*-*13* gene.

It was reported that only one patient from Ujiji had gametocytes up to day 2, possibly suggesting that patients were captured at early stage of the infection before developing falciparum gametocytes. On the other hand, microscopy could have failed to detect sub-microscopic gametocytes as previously shown [44]. Similar findings of gametocytes carriage among patients enrolled in clinical trials were reported in previous studies from Africa and elsewhere [45, 46]. However, the findings that only one patient from Ujiji had gametocytes in the first 3 days of the study was not expected and suggest that further assessment using more sensitive methods such as PCR may be needed, to confirm if most of febrile patients from this site do not carry gametocytes, suggesting that the transmission could possibly be driven and maintained by asymptomatic patients. A study conducted in Bagamoyo showed that asymptomatic individuals were more likely to carry gametocytes compared to symptomatic patients reporting to health facilities [44]. As also shown by other studies [47, 48], carriage of gametocytes by asymptomatic individuals could be critical in maintaining transmission in both malaria hyper and hypo-endemic areas suggesting that they should be targeted with transmission reducing interventions.

In this study, a progressive haematological improvement was observed from baseline to day 28 between the two sites and within the two treatment groups. However, patients from Ujiji had relatively higher Hb at enrolment which was maintained throughout the follow-up. Higher mean Hb at Ujiji than Muheza might be attributed to differences in nutritional status and other conditions associated with anaemia such as concurrent infections and helminth infestations [49–53]. It could also be due to differences in age, since patients enrolled at Ujiji had significantly higher mean age compared to Muheza (3.9 vs 3.2 years for Ujiji and Muheza, respectively). Improvements in Hb during follow-up could suggest that malaria might be a major contributing factor to the low haemoglobin levels and anaemia at recruitment as reported in other studies done in Tanzania [10] and elsewhere in sub-Saharan Africa [14, 15].

## Conclusion

The study showed that AL still remains highly efficacious for the treatment of uncomplicated falciparum malaria at the two sites and possibly in mainland Tanzania after its use for a decade. The study also revealed high efficacy of DP supporting the recommendations by NMCP to deploy it as an alternative ACT for improved case management in the country.

## Abbreviations

ACPR: adequate clinical and parasitological response
ACT: artemisinin-based combination therapy
AL: artemether–lumefantrine
AS + AQ: artesunate + amodiaquine
AS + MQ: artesunate + mefloquine
AS + SP: artesunate + sulfadoxine/pyrimethamine
DBS: dried blood spots on filter papers
DNA: deoxyribonucleic acid
DP: dihydroartemisinin–piperaquine
ETF: early treatment failure
*glurp*: glutamate-rich protein gene
Hb: haemoglobin
LCF: late clinical failure
LPF: late parasitological failure
MRCC: Medical Research Coordinating Committee
RDTs: malaria rapid diagnostic tests
*msp1*: merozoite surface protein-1 gene
*msp2*: merozoite surface protein-2 gene
NIMR: National Institute for Medical Research
NMCP: National Malaria Control Programme
OPD: outpatient department
PCR: polymerase chain reaction
PCT: parasite clearance time
SEA: Southeast Asia
WBCs: white blood cells
WHO: World Health Organization
